# Elevated O_3_ and TYLCV Infection Reduce the Suitability of Tomato as a Host for the Whitefly *Bemisia tabaci*

**DOI:** 10.3390/ijms17121964

**Published:** 2016-11-28

**Authors:** Hongying Cui, Yucheng Sun, Fajun Chen, Youjun Zhang, Feng Ge

**Affiliations:** 1 Department of Plant Protection, Institute of Vegetables and Flowers, Chinese Academy of Agricultural Sciences, Beijing 100081, China; cuihongying12345@163.com; 2 State Key Laboratory of Integrated Management of Pest Insects and Rodents, Institute of Zoology, Chinese Academy of Sciences, Beijing 100101, China; sunyc@ioz.ac.cn; 3 Department of Entomology, College of Plant Protection, Nanjing Agricultural University, Nanjing 210095, China; fajunchen@njau.edu.cn

**Keywords:** *Bemisia tabaci*, elevated O_3_, jasmonic acid, salicylic acid, *Tomato yellow leaf curl virus*

## Abstract

The effects of elevated atmospheric ozone (O_3_) levels on herbivorous insects have been well studied, but little is known about the combined effects of elevated O_3_ and virus infection on herbivorous insect performance. Using open-top chambers in the field, we determined the effects of elevated O_3_ and *Tomato yellow leaf curl virus* (TYLCV) infection on wild-type (Wt) tomato and *35S* tomato (jasmonic acid (JA) defense-enhanced genotype) in association with whitefly, *Bemisia tabaci* Gennadius biotype B. Elevated O_3_ and TYLCV infection, alone and in combination, significantly reduced the contents of soluble sugars and free amino acids, increased the contents of total phenolics and condensed tannins, and increased salicylic acid (SA) content and the expression of SA-related genes in leaves. The JA signaling pathway was upregulated by elevated O_3_, but downregulated by TYLCV infection and O_3_ + TYLCV infection. Regardless of plant genotype, elevated O_3_, TYLCV infection, or O_3_ + TYLCV infection significantly decreased *B. tabaci* fecundity and abundance. These results suggest that elevated O_3_ and TYLCV infection, alone and in combination, reduce the nutrients available for *B. tabaci*, increase SA content and SA-related gene expression, and increase secondary metabolites, resulting in decreases in fecundity and abundance of *B. tabaci* in both tomato genotypes.

## 1. Introduction

The concentration of global atmospheric ozone (O_3_) has increased from 10 parts per billion (ppb) in the 1900s to the current value of 40 ppb, at an annual rate of 1%–2% [1,2]. Moreover, the levels of atmospheric O_3_ are predicted to reach 68 ppb by the year 2050 [3]. The detrimental effects of O_3_ on plants have long been known. Elevated O_3_ causes leaf damage, inhibits photosynthesis, and reduces the growth of many plant species [4,5]. O_3_ enters the plant through stomata and is converted into reactive oxygen species (ROS), triggering a series of metabolic reactions [6,7]. Excess ROS can disrupt plant metabolism by causing irreversible damage to cell membranes, proteins, and carbohydrates [8]. Elevated O_3_ may change levels of primary metabolites and their allocation, leading to decreased nutrient content and increased levels of secondary metabolites in plant tissues [9,10]. Changes in the physical and chemical qualities of plant tissues are expected to affect herbivorous insects [11,12,13]. Furthermore, reduced plant quality is thought to be directly related to the virus susceptibility of plants grown in high-O_3_ environments [7,14].

Plant viruses can induce changes in their hosts that can affect the performance of herbivorous insects [15,16]. Viruses and other pathogens can alter plant photosynthesis, source/sink relationships, and defense responses [17]. For example, virus infection can activate or suppress plant defense pathways, such as the salicylic acid (SA) and jasmonic acid (JA) pathways [18,19]. Increasing evidence suggests that vector-borne pathogens can alter the quality of their hosts in ways that influence the abundance of herbivores [15,16,20]. Although several studies have documented how changes in nutrients or resistance of virus-infected plants affect the performance of herbivorous insects [20,21], little is known about how the combined effects of changes in nutrients and resistance caused by virus infection affect the performance of herbivorous insects. 

Both plant viruses and elevated O_3_ can induce hormone-mediated resistance that, in turn, affects herbivorous insects [10,21]. SA and JA are regarded as the most important hormonal mediators of induced defenses of plants against pathogens, ozone, herbivores, and other stressors [22,23,24,25]. The prevailing view is that the SA pathway induces resistance against biotrophic pathogens and some phloem feeders, whereas the JA pathway induces resistance against chewing herbivores and necrotrophic pathogens [25,26]. Crosstalk between SA and JA signaling pathways may mediate the reciprocal effects of induced plant defenses on pathogens and herbivores [27,28,29]. Plant hormones interact at many different levels to form a network of antagonistic and synergistic interactions [25,30]. For example, SA accumulation in plant tissues is often negatively correlated with JA accumulation [31,32] and can thus suppress the induction of JA-mediated defenses [33,34,35]. Thus, infection by *Tomato yellow leaf curl virus* (TYLCV) increases the SA level and suppresses the JA level in tomato [36]. On the other hand, elevated O_3_ induced the accumulation of both JA and SA [37,38,39]. Previous studies showed that elevated CO_2_ altered the cross talk between the SA and JA defense pathways following TYLCV infection, i.e., the interactions between the pathways were antagonistic (when one rises, the other falls) under ambient CO_2_, but synergistic under elevated CO_2_ [40]. Whether elevated O_3_ concentrations alter the interactions between SA- and JA-dependent defense pathways following TYLCV infection is unknown. 

TYLCV is transmitted by the whitefly *Bemisia tabaci* (Hemiptera: Aleyrodidae) in a persistent circulative manner. This virus has devastated tomato production in a part of China and is frequently found on tomatoes in areas where *B. tabaci* occurs [21,41]. TYLCV disease outbreaks have also occurred worldwide and are thought to be related to global climate change [42]. *B. tabaci* is an invasive phloem pest with a worldwide distribution [43]. Whiteflies puncture leaf tissue with piercing-sucking mouthparts and feed on the phloem [44]. *B. tabaci* has been particularly damaging to tomato crops [45,46] and especially in China [47]. Tomato (*Lycopersicon esculentum*) is an economically important crop worldwide and is sensitive to O_3_ [48]. Little is known about the interactive effects of elevated O_3_ and TYLCV infection on the performance of *B. tabaci* on tomato.

JA acts as a signaling molecule for the production of metabolites that contribute to resistance [49]. Instead of JA-dependent defenses, phloem-feeding insects trigger SA-dependent defenses, which could avoid strong resistance. In previous studies, JA accumulation was increased by elevated O_3_ but suppressed by TYLCV [21,39]. Rather than being independent, JA and SA interact with each other in response to abiotic and biotic factors [22,23,50]. Application of exogenous JA to plants results in an increase in the production of a diverse array of compounds that have been shown to reduce the performance of herbivores [51,52]. However, the effect of endogenous JA on the performance of whiteflies on plants exposed to elevated O_3_ and TYLCV infection is unclear. The JA defense-enhanced tomato genotype *35S* has a stronger JA signal and greater resistance than the wild-type (Wt), but how the endogenously high levels of JA in *35S* plants affect *B. tabaci* and TYLCV is unclear. Here, we tested the hypothesis that elevated O_3_ and TYLCV infection will decrease the fitness of *B. tabaci* by altering the nutrient content and resistance of *35S* and Wt tomato plants. Our specific objectives were to determine the effects of elevated O_3_ and TYLCV infection alone and in combination on the nutrient content, resistance of tomato, and the performance of *B. tabaci*.

## 2. Results

### 2.1. Tomato Growth Traits

Both O_3_ and TYLCV decreased plant biomass and height independently, and together biomass and height were even lower. The response differed between the two tomato genotypes leading to a significant three-way interaction among the treatments (Table 1).

**Figure 1:**
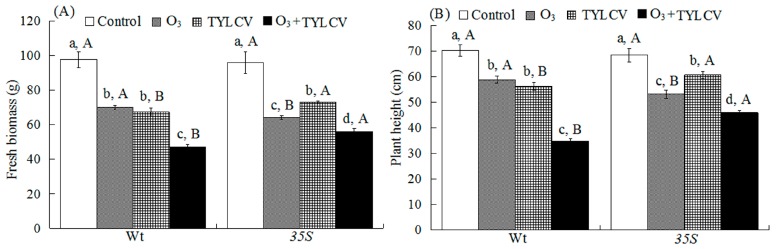
Fresh biomass (**A**); and plant height (**B**) of two tomato genotypes (Wt and *35S*) grown under ambient and elevated O_3_ with and without TYLCV infection for six weeks. Control refers to plants grown under ambient O_3_ and without TYLCV. O_3_ refers to uninfected plants grown under elevated O_3_. TYLCV refers to TYLCV-infected plants grown under ambient O_3_. O_3_ + TYLCV refers to TYLCV-infected plants grown under elevated O_3_. Each value represents the average (±SE) of 24 replicates. Different lowercase letters within a row indicate significant differences among the four treatments in a specific tomato cultivar, and different uppercase letters indicate significant differences between the two tomato genotypes within the same treatment (Tukey’s test: *p* < 0.05).

In the Wt genotype, elevated O_3_ decreased fresh weight by 28% for uninfected plants and by 52% for TYLCV-infected plants, and decreased height by 16% for uninfected plants and by 50% for TYLCV-infected plants. In the *35S* genotype, elevated O_3_ decreased fresh weight by 33% for uninfected plants and by 41% for TYLCV-infected plants, and decreased height by 22% for uninfected plants and by 33% for TYLCV-infected plants. Regardless of O_3_ level, TYLCV infection significantly decreased the fresh weight and height of the two tomato genotypes. Moreover, both of them were the lowest under O_3_ + TYLCV infection treatment on the two tomato genotypes. Wt plants had higher fresh weight and height than *35S* plants for the treatment of elevated O_3_, but had lower fresh weight and height than *35S* plants for the treatments of TYLCV infection and O_3_ + TYLCV (Figure 1A,B).

### 2.2. Foliar Soluble Sugar and Free Amino Acids of Tomato

Both O_3_ and TYLCV decreased soluble sugar content independently, and together soluble sugar content was even lower (Table 2). Both O_3_ and TYLCV decreased free amino acid content independently, and together free amino acid content was even lower. The response differed between the two tomato genotypes leading to a significant three-way interaction among the treatments (Table 2).

**Figure 2:**
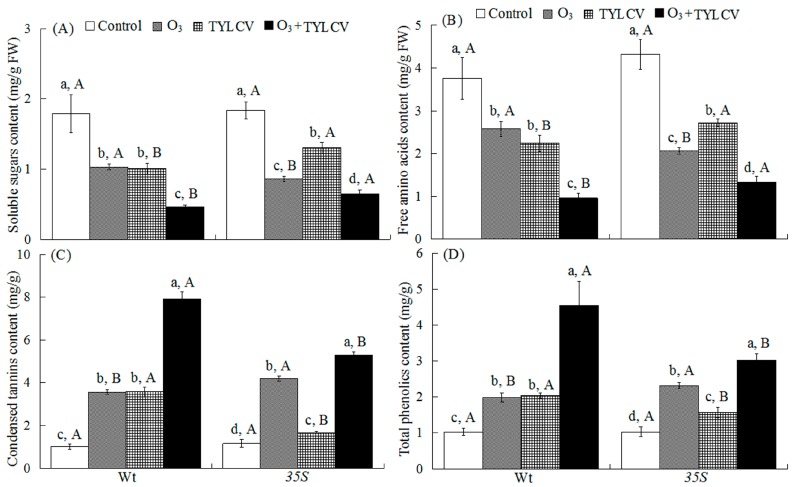
Concentrations of soluble sugars (**A**); free amino acids (**B**); condensed tannins (**C**); and total phenolics (**D**) in the two tomato genotypes (Wt and *35S*) grown under ambient and elevated O_3_ with and without TYLCV infection after six weeks. Treatments are explained in Figure 1. Each value represents the average (±SE) of four replicates. Different lowercase letters within a row indicate significant differences among the four treatments in a specific tomato cultivar, and different uppercase letters indicate significant differences between tomato genotypes within the same treatment (Tukey’s test: *p* < 0.05).

In the Wt genotype, elevated O_3_ decreased soluble sugar content by 42% for uninfected plants and by 74% for TYLCV-infected plants, and decreased free amino acid content by 31% for uninfected plants and by 74% for TYLCV-infected plants. In the *35S* genotype, elevated O_3_ decreased soluble sugar content by 53% for uninfected plants and by 65% for TYLCV-infected plants, and decreased free amino acid content by 52% for uninfected plants and by 69% for TYLCV-infected plants. Regardless of O_3_ level, TYLCV infection significantly decreased soluble sugar and free amino acid contents in both tomato genotypes. Moreover, soluble sugar and free amino acid contents were the lowest with O_3_ + TYLCV infection treatment for both genotypes. Wt plants had higher soluble sugar and free amino acid contents than *35S* plants for the treatment of elevated O_3_, but had lower soluble sugar and free amino acid contents than *35S* plants for the treatments of TYLCV infection and O_3_ + TYLCV (Figure 2A,B). 

### 2.3. Condensed Tannins and Total Phenolics in Tomato Leaves

Both O_3_ and TYLCV increased the contents of condensed tannins and total phenolics independently, and together they were even higher. The response differed between the two tomato genotypes leading to a significant three-way interaction among the treatments (Table 2). In the Wt genotype, elevated O_3_ increased condensed tannin content 2.5-fold for uninfected plants and 6.8-fold for TYLCV-infected plants, and increased total phenolics content 92% for uninfected plants and 3.4-fold for TYLCV-infected plants. In the *35S* genotype, elevated O_3_ increased condensed tannin content 2.6-fold for uninfected plants and 3.6-fold for TYLCV-infected plants, and increased total phenolics content 1.2-fold for uninfected plants and 1.9-fold for TYLCV-infected plants. Regardless of O_3_ level, TYLCV infection significantly increased the contents of condensed tannins and total phenolics in both genotypes. Both condensed tannins and total phenolics were highest in O_3_ + TYLCV infection treatment for both genotypes. Wt plants had lower condensed tannins and total phenolics contents than *35S* plants for the treatment of elevated O_3_, but had higher condensed tannins and total phenolics contents than *35S* plants for the treatments of TYLCV infection and O_3_ + TYLCV (Figure 2C,D).

### 2.4. SA Content and Expression of Phenylalanine Ammonia Lyase Gene (PAL) and Pathogenesis-Related Protein Gene (PR1) in Tomato

**Figure 3:**
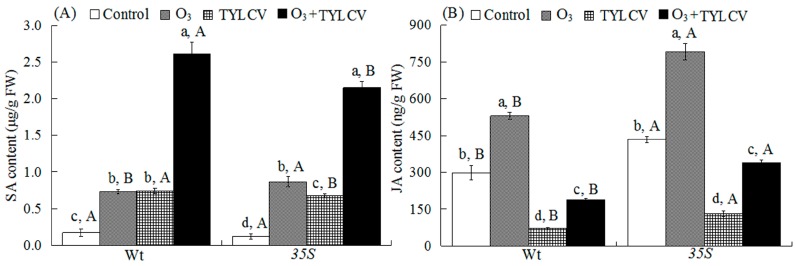
Concentrations of (**A**) salicylic acid (SA); and (**B**) jasmonic acid (JA) in the two tomato genotypes (Wt and *35S*) grown under ambient and elevated O_3_ with and without TYLCV infection after six weeks. Treatments are explained in Figure 1. Each value represents the average (±SE) of four replicates. Different lowercase letters within a row indicate significant differences among the four treatments in a specific tomato cultivar, and different uppercase letters indicate significant differences between the two tomato genotypes within the same treatment (Tukey’s test: *p* < 0.05).

**Figure 4:**
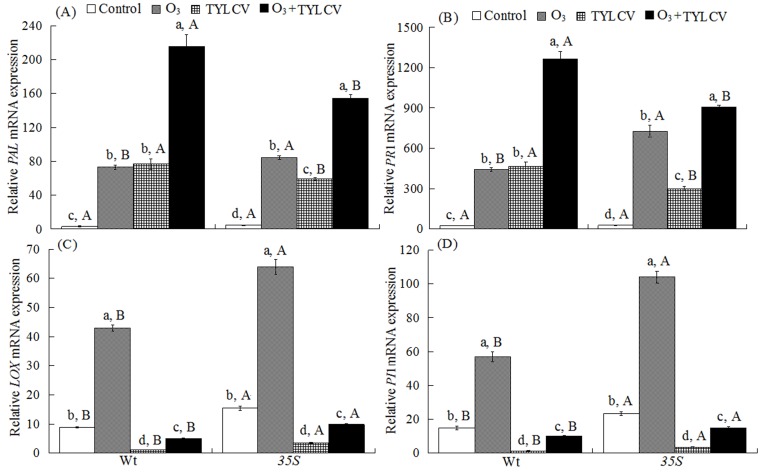
The relative expression of genes encoding (**A**) phenylalanine ammonia lyase (*PAL*); (**B**) pathogenesis-related protein (*PR1*); (**C**) lipoxygenases (*LOX*); and (**D**) proteinase inhibitor (*PI1*) in the two tomato genotypes (Wt and *35S*) grown under ambient and elevated O_3_ with and without TYLCV infection after six weeks. Treatments are explained in Figure 1. Each value represents the average (±SE) of four replicates. Different lowercase letters within a row indicate significant differences among the four treatments in a specific tomato cultivar, and different uppercase letters indicate significant differences between the two tomato genotypes within the same treatment (Tukey’s test: *p* < 0.05).

Both O_3_ and TYLCV increased SA content and the relative expression of *PAL* and *PR1* mRNA independently, and together they were even higher. The response differed between the two tomato genotypes leading to a significant three-way interaction among the treatments (Table 2). For uninfected plants, elevated O_3_ increased the SA content and the relative expression of *PAL* and *PR1* mRNA 3.3-fold, 22.6-fold, and 18.1-fold, respectively, in the Wt genotype, and by 6.2-fold, 17.6-fold, and 26.5-fold, respectively, in the *35S* genotype. For TYLCV-infected plants, elevated O_3_ increased the SA content and the relative expression of *PAL* and *PR1* mRNA by 14.2-fold, 68.5-fold, and 53.8-fold, respectively, in the Wt genotype, and by 16.9-fold, 33.1-fold, and 33.3-fold, respectively, in the *35S* genotype. Regardless of O_3_ level, TYLCV infection significantly increased the SA content and relative expression of *PAL* and *PR1* mRNA in both genotypes. The SA content and relative expression of *PAL* and *PR1* mRNA were highest with O_3_ + TYLCV infection treatment for both genotypes. Wt plants had lower SA content and relative expression of *PAL* and *PR1* mRNA than *35S* plants for the treatment of elevated O_3_, but had higher SA content and relative expression of *PAL* and *PR1* mRNA than *35S* plants for the treatments of TYLCV infection and O_3_ + TYLCV (Figure 3A and Figure 4A,B).

### 2.5. JA Content and Expression of Lipoxygenase Gene (LOX) and Proteinase Inhibitor Gene (PI1) in Tomato

O_3_ increased JA content and the relative expression of *LOX* and *PI1* mRNA, while TYLCV decreased them significantly. The response differed between the two tomato genotypes leading to a significant three-way interaction among the treatments on the relative expression of *LOX* and *PI1* mRNA (Table 2).

In uninfected plants, elevated O_3_ increased the JA content and the relative expression of *LOX* and *PI1* mRNA by 78%, 3.9-fold, and 2.8-fold, respectively, in the Wt plants, and by 82%, 3.1-fold, and 3.5-fold, respectively, in the *35S* plants. In TYLCV-infected plants, elevated O_3_ decreased the JA content and the relative expression of *LOX* and *PI1* mRNA by 37%, 44%, and 34%, respectively, in the Wt genotype, and by 22%, 37%, and 36%, respectively, in the *35S* genotype. Regardless of O_3_ level, TYLCV infection significantly decreased the JA content and the relative expression of *LOX* and *PI1* mRNA in both genotypes. The JA content and the relative expression of *LOX* and *PI1* mRNA were lower in Wt plants than in *35S* plants in the control (ambient O_3_ and no TYLCV infection) and in the elevated O_3_, TYLCV infection, and O_3_ + TYLCV infection treatments (Figure 3B and Figure 4C,D).

### 2.6. Fecundity and Abundance of B. tabaci

Both O_3_ and TYLCV decreased *B. tabaci* fecundity and abundance independently, and together fecundity and abundance were even lower (Table 1).

**Figure 5:**
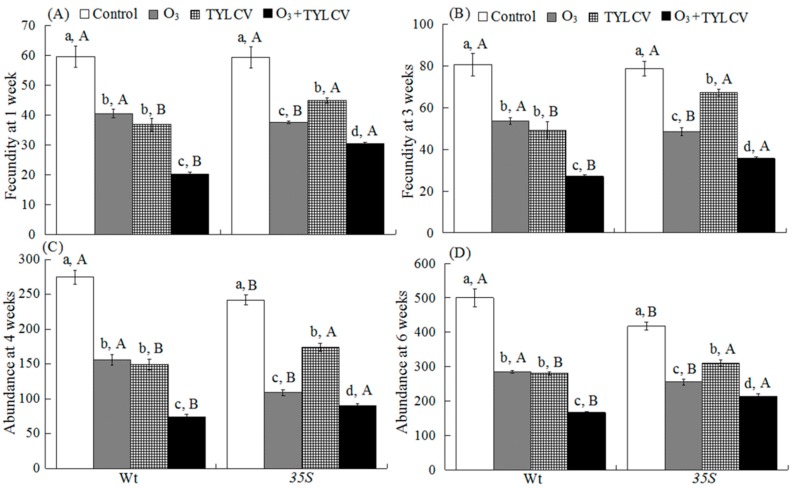
*B. tabaci* fecundity (**A**) at one week; and (**B**) at three weeks; and *B. tabaci* numbers per plant (**C**) at four weeks; and (**D**) at six weeks on two tomato genotypes (Wt and *35S*) grown under ambient and elevated O_3_ with and without TYLCV infection. Treatments are explained in Figure 1. Each value represents the average (±SE) of 24 replicates. Different lowercase letters within a row indicate significant differences among the four treatments in a specific tomato cultivar, and different uppercase letters indicate significant differences between the two tomato genotypes within the same treatment (Tukey’s test: *p* < 0.05). “Weeks” refer to the start of the oviposition period of the single insect pairs (that occurred three weeks, four weeks and six weeks after the beginning of the experiment).

Elevated O_3_ decreased fecundity by 32% (19) at one week and by 34% at three weeks (27) on uninfected Wt plants, and by 37% (22) at one week and by 39% (30) at three weeks on uninfected *35S* plants. Elevated O_3_ decreased fecundity by 66% (39) at one week and by 67% (53) at three weeks on TYLCV-infected Wt plants, and by 49% (29) at one week and by 55% (43) at three weeks on TYLCV-infected *35S* plants (Figure 5A,B). Elevated O_3_ decreased abundance by 43% (119) at four weeks and by 44% (215) at six weeks on uninfected Wt plants, and by 55% (132) at four weeks and by 39% (161) at six weeks on uninfected *35S* plants. Elevated O_3_ decreased abundance by 73% (200) at four weeks and by 66% (333) at six weeks on TYLCV-infected Wt plants, and by 64% (153) at four weeks and by 49% (203) at six weeks on TYLCV-infected *35S* plants (Figure 5C,D).

Regardless of O_3_ level, TYLCV infection significantly decreased *B. tabaci* fecundity and abundance on both genotypes. Fecundity and abundance were lowest with the O_3_ + TYLCV infection treatment on both genotypes (Figure 5A–D). Fecundity was higher on Wt plants than on *35S* plants in the elevated O_3_ treatment, but was lower in the TYLCV infection and O_3_ + TYLCV treatments (Figure 5A,B). Abundance was higher on Wt plants than on *35S* plants in the control and the elevated O_3_ treatments, but was lower in the TYLCV infection and O_3_ + TYLCV treatments (Figure 5C,D).

### 2.7. Pearson Correlations between B. tabaci Fecundity and Abundance and Biochemical Properties of Tomato Leaves

*B. tabaci* fecundity and abundance were positively correlated with the contents of soluble sugars and free amino acids in tomato leaves (Table 3). *B. tabaci* fecundity and abundance were negatively correlated with the contents of condensed tannins, total phenolics, and SA, and with the relative expression of *PAL* and *PR1* mRNA (Table 3). 

## 3. Discussion

Atmospheric ozone concentrations are likely to increase in the future and are likely to alter the occurrences of diseases and insect pests [10,39,53]. Although much is known about the effects of elevated O_3_ on viruses and insect pests, little is known about the effects of elevated O_3_ and virus-infected plant on the performance of insect pests. O_3_ is highly phytotoxic [54,55,56,57]. In the current study, elevated O_3_ significantly reduced the fresh biomass and height of Wt tomato plants and also reduced their free amino acid and soluble sugar contents. In previous studies, elevated O_3_ decreased concentrations of carbohydrates and nutrients in plants [9,10]. Such reductions are likely to affect whiteflies because phloem-sucking insects are adversely affected by low levels of available amino acids [10,58] and by low levels of soluble sugars [59,60] in host plants.

In the present study, elevated O_3_ significantly increased the contents of secondary metabolites, SA, and JA, and the expression of SA- and JA-related genes in Wt tomato plants. The *PAL* gene plays a key role in SA biosynthesis and in the regulation of synthesis in secondary metabolism, while the *PR1* transcript is a marker for SA response [61,62,63]. The JA-responsive upstream gene *LOX* and downstream gene *PI1* are important in the JA signaling pathway [21]. Elevated SA levels can decrease aphid abundance [13,64]. *PR1* and *PI1* proteins have been shown to increase plant resistance against aphids and whiteflies [21,65,66]. Secondary metabolites have been found to decrease the *r_m_* values and the population densities of phloem-feeding insects [60,67,68]. Our results also showed that elevated O_3_ significantly decreased *B. tabaci* fecundity and abundance. *B. tabaci* fecundity and abundance were positively correlated with plant nutrient content but negatively correlated with secondary metabolite content, SA content, and SA-related gene expression. These results suggest that elevated O_3_ significantly reduces the nutrient content and increases the resistance of tomato plants, which, together, result in a decrease in *B. tabaci* fecundity and abundance. 

The current results showed that TYLCV infection significantly reduced the fresh biomass and height of Wt tomato plants. Previous studies found that TYLCV infection reduced the amino acid content of tomato leaves [69]. Our results showed that TYLCV also reduced nutrient levels in Wt tomato plants. A recent report showed that *Tomato spotted wilt virus* (TSWV) increased SA levels and SA-related marker gene expression, but reduced JA content and JA-related gene expression in *Arabidopsis* plants [70]. We similarly found that TYLCV infection significantly increased secondary metabolites, SA content, and SA-related gene expression, but decreased JA content and JA-related gene expression in Wt tomato plants. TYLCV infection also decreased *B. tabaci* fecundity and abundance. These results suggest that by reducing the nutrient content and by increasing the SA content and the expression of SA-related genes (while not increasing JA content or the expression of JA-related genes) in tomato plants, TYLCV infection decreases the fecundity and abundance of *B. tabaci*. 

The fresh biomass and height of Wt tomato plants were much lower in the O_3_ + TYLCV infection treatment than in the control, elevated O_3_, and TYLCV infection treatments. Reductions in biomass and height are among the important symptoms caused by plant viruses [71,72]. In the current study, elevated O_3_ levels significantly reduced biomass and height of TYLCV-infected tomato plants. This suggests that yield losses caused by TYLCV on tomato will increase if atmospheric O_3_ levels continue to increase. The contents of free amino acids and soluble sugars in Wt tomato plants were much lower in the O_3_ + TYLCV infection treatment than in the control, elevated O_3_, or TYLCV infection treatments, suggesting that elevated O_3_ significantly reduce TYLCV-infected tomato plant nutrients. SA content and SA-related gene expression were higher in the O_3_ + TYLCV infection than in the other treatments. However, JA content and JA-related gene expression were higher in the O_3_ + TYLCV infection treatment than in the TYLCV infection treatment but were lower in the O_3_ + TYLCV infection treatment than in the control or elevated O_3_ treatment. This suggests that elevated O_3_ and TYLCV infection together further enhance the SA pathway but suppress the JA pathway. *B. tabaci* fecundity and abundance were lowest in the O_3_ + TYLCV infection treatment. The results suggest that elevated O_3_ further suppress *B. tabaci* fecundity and abundance on TYLCV-infected tomato plants.

Several JA-overexpression mutants exhibit increased resistance against insects [73,74,75]. The performance of the pea leaf miner, the root-knot nematode, and *B. tabaci* differed on JA-overexpression *35S* tomato plants than on Wt tomato plants [10,76,77]. In the current study, the fresh weight and height were lower for *35S* plants than for Wt plant in the elevated O_3_ treatment but were higher in the TYLCV infection and O_3_ + TYLCV infection treatments. Assuming that O_3_ levels continue to increase, these results suggest that *35S* plants may perform better than Wt plants when infected with TYLCV. The nutrient levels were lower in *35S* plants than in Wt plants in the elevated O_3_ treatment but were higher in the TYLCV infection and O_3_ + TYLCV infection treatments. At the same time, secondary metabolite content, SA content, and the expression of SA-related genes were higher in *35S* plants than in Wt plants in the elevated O_3_ treatment, but were lower in *35S* plants than in Wt plants in the TYLCV infection and O_3_ + TYLCV infection treatments. This resulted in lower *B. tabaci* fecundity and abundance on *35S* plants than on Wt plant with the elevated O_3_ treatment, but higher fecundity and abundance on *35S* plants than on Wt plants with the TYLCV infection and O_3_ + TYLCV infection treatments. These results indicate that the JA-overexpression tomato mutant *35S* has higher resistance to *B. tabaci* than Wt plants under elevated O_3_, and that resistance to *B. tabaci* is decreased by TYLCV infection of *35S* plants. Wt plants exhibit greater resistance to *B. tabaci* than *35S* plants in the TYLCV infection and O_3_ + TYLCV infection treatments. Furthermore, *B. tabaci* abundance was lower on *35S* plants than on Wt plants in the control. JA content and JA-related gene expression were higher in *35S* plants than in Wt plants in the control, while SA content and SA-related gene expression did not differ between the two genotypes in the control. This suggests that the JA pathway and JA-related gene expression also participate in the deterring *B. tabaci* performance.

Mutual antagonism between the SA and JA pathways has been well documented [78,79], but evidence of synergistic interactions have also been reported [80,81]. In this study, elevated O_3_ increased the levels of SA and JA, while TYLCV infection increased the SA level and reduced the JA level. Moreover, elevated O_3_ increased the SA level and reduced the JA level after TYLCV infection. This suggests that TYLCV infection alters the interactions between SA and JA pathways under elevated O_3_. Previous studies showed that the combined application of exogenous SA and JA induced stronger resistance against TYLCV than application of either SA or JA alone [40]. Overall, the results suggest that the altered interaction between SA and JA under elevated O_3_ will increase TYLCV incidence and severity as atmospheric O_3_ levels continue to increase. 

Our results showed that elevated O_3_ significantly decreased *B. tabaci* fecundity and abundance which was beneficial to plant growth/yield. However, elevated O_3_ significantly reduced the fresh biomass of tomato plants. It is worthwhile to evaluate the plant yield losses in future studies when insect pest and pathogen are considered under an elevated O_3_ environment. 

## 4. Materials and Methods

### 4.1. Open-Top Chambers

The experiment was conducted using eight octagonal open-top chambers (OTCs) at the Observation Station for Global Change Biology, the Institute of Zoology of the Chinese Academy of Sciences in Xiaotangshan County, Beijing, China (40°11′ N, 116°24′ E). Each OTC was 2.2 m in height and 2 m in diameter. The O_3_ levels were increased in four of the OTCs but were kept at ambient levels in the other four beginning on 28 June 2014. A detailed description of the O_3_ system was provided by Cui et al. [82]. O_3_ levels were measured hourly and averaged 37.3 ppb in the ambient OTCs and 72.2 ppb in the elevated OTCs. The OTCs were ventilated with air daily from 8:00 a.m. to 6:00 p.m. The experiment was terminated after six weeks on 13 August 2014. We measured the air temperature three times daily (08:00, 14:00, 18:00) throughout the experiment, and there is no significant difference between the two sets of OTCs (28.05 ± 3.07 °C in ambient O_3_ chambers and 28.65 ± 3.67 °C in elevated O_3_ chambers in the year of 2014). 

### 4.2. Host Plants

The *35S*::*prosystemin* transgenic tomato plants (*35S*), and its background wild-type (Wt) tomato plants (*Solanum esculentum* cv. Castlemart) were individually transplanted into plastic pots (14 cm diameter, 12 cm height) filled with sterilized loamy soil after two weeks of growth in sterilized soil. Since over-expression of prosystemin, the *35S* transgenic plants constitutively activates defenses in unwounded plants, which leads to a stronger and faster defense [75]. Plants at the three to four leaf stage were placed in ventilated cages in the OTCs on 27 June 2014. Each ventilated cage (1.0 m long, 1.0 m wide, 1.8 m high, 80 mesh) contained 24 seedlings (12 individuals from each tomato genotype including six plants for growth traits, the other six plants for *B. tabaci* fecundity and abundance), and two ventilated cages were placed in each of the eight OTCs. 48 plants were transferred to each OTC and randomly split into each cage. Twenty-four plants in each cage were inoculated with TYLCV, while the others were inoculated with LB culture medium as a control.

### 4.3. Tomato yellow leaf curl virus Clone and Agroinoculation

One day after they were placed in the OTCs, the designated tomato plants were infected with TYLCV via *Agrobacterium tumefaciens*-mediated inoculation [34,83]. The method of TYLCV clone and inoculation mainly follows that described as Huang et al. [40]. 

### 4.4. Tomato Growth Traits

The growth traits of the tomato plants in the OTCs were assessed when the experiment was terminated. Plant height and fresh biomass were determined for uninfected and infected plants grown in 37.3 ppb and 72.2 ppb O_3_. Six plants of each genotype (6 × 4 OTCs = 24 plant in total) in each OTC were selected for determination of plant height and fresh biomass, and harvested for analysis of biochemical and genic parameters. They were described and assayed in the following paragraphs.

### 4.5. Tomato Foliar Chemistry

The contents of free amino acids, soluble sugars, total phenolics, and condensed tannins in tomato leaves were measured according to Cui et al. [10]. For measurement of SA and JA contents, approximately 500 mg of leaf tissue (fresh weight) was extracted for SA and JA quantification as described previously [10]. For determination of the relative expression of *PR1*, *PAL*, *PI1*, and *LOX* mRNA, a sample of fresh leaves from each plant was removed and stored at −78 °C for real-time PCR following the procedures described by Sun et al. [77]. Each treatment combination was represented by four biological repeats, and each biological repeat had three technical repeats. Real-time quantitative PCR was used to quantify the mRNAs of the *PR1*, *PAL*, *PI1*, and *LOX*. Primer pairs for qRT-PCR are listed in Supplementary Material Table S1. A detailed description of the quantification (*PR1*, *PAL*, *PI1*, and *LOX*) was provided by Sun et al. [77].

### 4.6. Fecundity and Abundance of B. tabaci

The B biotype of *B. tabaci* was collected from cabbage growing at the Beijing Academy of Agriculture and Forestry on 19 March 2014. The biotype was determined by assessing amplified fragment-length polymorphism (AFLP) markers [84], and the population was reared on tomatoes in a greenhouse. To determine the effects of O_3_ level, cultivar, and TYLCV infection on *B. tabaci* fecundity, tomato plants of uniform size and of each cultivar were randomly selected to each OTC (32 individuals from each tomato genotype × two genotypes). Three weeks after the start of experiment (about three weeks after virus inoculation), one clip-cage (3.5 cm diameter, 1.5 cm height) was attached to each of three leaves on each plant, and one newly-emerged adult female and one newly-emerged adult male of *B. tabaci* were placed in each cage. After one week, the eggs in each cage were counted, and the clip-cage and adults were moved to a new leaf. After two additional weeks, the eggs (including hatched and unhatched) on the new leaves were counted. If a male died, another healthy male was selected and immediately added until the female died. We checked the survivals for each pair of whitefly daily. Fecundity was recorded as the total number of eggs produced by one pair of whiteflies. 

To determine the effects of O_3_ level, cultivar, and TYLCV infection on *B. tabaci* abundance, tomato plants of uniform size and of each cultivar were randomly selected to each OTC (64 individuals from each tomato genotype × two genotypes). One week after the start of experiment, one clip-cage (9 cm diameter, 4 cm height) was attached to each of three leaves on each plant, and four newly-emerged female adults and four newly-emerged male adults of *B. tabaci* were placed in each cage. After three and five additional weeks, the number of *B. tabaci* in each cage was determined, and abundance was recorded as the total number (eggs, nymphs and adults) per plant.

### 4.7. Statistical Analyses

The experiment had a split-split plot design with O_3_ and block (a pair of ambient and elevated OTCs) as the main effects, TYLCV as the subplot effect, and tomato genotypes as the sub-subplot effect. The main effects of O_3_, TYLCV, and tomato genotype on plant and *B. tabaci* variables were tested according to the following model (ANOVA, PASW, 2009):

*X_ijklm_* = μ + O*_i_* + *B*(O)_*j*(*i*)_ + V*_k_* + OV*_ik_* + VB(O)_*kj*(*i*)_ + T*_l_* + OT*_il_* + TB(O)_*lj*(*i*)_ + VTB(O)_*klj*(*i*)_ + e_*m*(*ijkl*)_

where O is the O_3_ treatment (*i* = 2), *B* is the block (*j* = 4), V is the TYLCV treatment (*k* = 2), and T is the tomato genotype (*l* = 2). *X_ijklm_* represents the error because of the smaller scale differences between samples and variability within blocks (SPSS 13.0, SPSS lnc., Chicago, IL, USA). Tukey’s multiple range tests were used to separate means when ANOVAs were significant (*p* < 0.05). Pearson’s correlations were calculated to analyze the relationships between the fecundity and abundance of *B. tabaci* and the soluble sugars, free amino acids, SA levels, relative expression of *PR1* and *PAL* mRNA, total phenolics, and the condensed tannin content of tomatoes grown with all eight combinations of treatments.

## 5. Conclusions

Our results indicate that elevated O_3_ and TYLCV infection, alone and in combination, significantly reduce the nutrient content of tomato plants and increase SA levels, the relative expression of *PR1* and *PAL* mRNA, and secondary metabolite levels, which, together, decrease *B. tabaci* fecundity and abundance on two tomato genotypes. Furthermore, elevated O_3_ levels significantly reduced *B. tabaci* abundance on TYLCV-infected tomato plants. Such changes suggest that the carrying capacity of the environment with respect to *B. tabaci* will decrease with the increases in O_3_ levels and TYLCV infection. *35S* plants have higher resistance to *B. tabaci* than Wt plants under elevated O_3_, but Wt plants have higher resistance to *B. tabaci* than *35S* plants when the plants are infected with TYLCV or when the plants are infected with TYLCV and grown under elevated O_3_. These results should assist in the development of cultivars that are resistant to increasing O_3_ levels, TYLCV, and *B. tabaci*.

## Figures and Tables

**Table 1:** Effects of O_3_ level, TYLCV infection, and plant genotype on *B. tabaci* fecundity (egg/pair) and abundance (number/plant), plant fresh biomass, plant height of tomato. *F* and *p* values from ANOVA are shown.

Measured Indices	Value	Treatment_(df)_						
		O_3 (1, 184)_	TYLCV _(1, 184)_	Tomato Genotype _(1, 184)_	O_3_ × TYLCV _(1, 184)_	O_3_ × Genotype _(1, 184)_	TYLCV × Genotype _(1, 184)_	O_3_ × TYLCV × Genotype _(1, 184)_
Fresh biomass	*F*	930.88	703.90	4.72	50.27	0.06	50.27	4.77
	*p*	0.00	0.00	0.03	0.00	0.80	0.00	0.03
Plant height	*F*	297.10	212.51	5.05	6.83	0.61	40.02	8.61
	*p*	0.00	0.00	0.03	0.01	0.44	0.00	0.00
Fecundity at one week	*F*	164.26	132.49	6.86	14.61	0.02	3.04	0.75
	*p*	0.00	0.00	0.01	0.00	0.90	0.08	0.39
Fecundity at three weeks	*F*	164.52	90.42	5.14	15.33	2.15	0.19	0.58
	*p*	0.00	0.00	0.03	0.00	0.14	0.67	0.45
Abundance at four weeks	*F*	1768.14	908.72	16.88	89.48	5.49	151.95	0.33
	*p*	0.00	0.00	0.00	0.00	0.02	0.00	0.57
Abundance at six weeks	*F*	1246.29	851.42	4.12	100.69	18.25	131.01	0.30
	*p*	0.00	0.00	0.04	0.00	0.00	0.00	0.58

“Weeks” refer to the start of the oviposition period of the single insect pairs (that occurred three weeks, four weeks and six weeks after the beginning of the experiment).

**Table 2:** Effects of O_3_ level, TYLCV infection, and plant genotype on biochemical properties of tomato. *F* and *p* values from ANOVA are shown.

Measured Indices	Value	Treatment_(df)_						
		O_3 (1, 24)_	TYLCV _(1, 24)_	Tomato Genotype _(1, 24)_	O_3_ × TYLCV _(1, 24)_	O_3_ × Genotype _(1, 24)_	TYLCV × Genotype _(1, 24)_	O_3_ × TYLCV × Genotype _(1, 24)_
Soluble sugars	*F*	307.79	156.07	4.89	9.85	4.01	12.86	0.56
	*p*	0.00	0.00	0.03	0.00	0.07	0.00	0.46
Free amino acids	*F*	297.79	238.19	6.47	4.80	11.22	5.11	7.75
	*p*	0.00	0.00	0.02	0.04	0.00	0.03	0.01
Condensed tannins	*F*	681.27	269.61	52.13	21.25	0.18	104.37	5.37
	*p*	0.00	0.00	0.00	0.00	0.68	0.00	0.03
Total phenolics	*F*	159.07	96.41	11.39	12.30	2.04	22.38	7.74
	*p*	0.00	0.00	0.00	0.00	0.17	0.00	0.01
SA ^a^	*F*	590.53	503.75	4.42	103.99	1.83	11.67	7.68
	*p*	0.00	0.00	0.04	0.00	0.19	0.00	0.01
JA ^b^	*F*	304.52	641.86	136.01	26.12	17.05	12.89	0.48
	*p*	0.00	0.00	0.00	0.00	0.00	0.00	0.50
*PAL* ^c^	*F*	515.41	406.44	15.52	24.91	4.15	29.07	9.94
	*p*	0.00	0.00	0.00	0.00	0.05	0.00	0.00
*PR1* ^d^	*F*	926.49	428.75	8.23	12.23	1.15	97.03	33.08
	*p*	0.00	0.00	0.01	0.00	0.29	0.00	0.00
*LOX* ^e^	*F*	984.33	1430.40	139.61	601.29	32.77	47.78	16.62
	*p*	0.00	0.00	0.00	0.00	0.00	0.00	0.00
*PI1* ^f^	*F*	829.22	1170.85	160.05	428.72	70.30	94.43	53.38
	*p*	0.00	0.00	0.00	0.00	0.00	0.00	0.00

^a^ Salicylic acid; ^b^ Jasmonic acid; ^c^ Phenylalanine ammonia lyase; ^d^ Pathogenesis-related protein; ^e^ Lipoxygenases; ^f^ Proteinase inhibitor.

**Table 3:** Pearson correlations between *B. tabaci* fecundity (egg/pair) and abundance (number/plant) and biochemical properties of tomato leaves.

Tomato Constituents	Fecundity at One Week			Fecundity at Three Weeks			Abundance at Four Weeks			Abundance at Six Weeks		
	df	*r*	*p*	df	*r*	*p*	df	*r*	*p*	df	*r*	*p*
Soluble sugars	6	0.995	0.000	6	0.989	0.000	6	0.981	0.000	6	0.970	0.000
Free amino acids	6	0.984	0.000	6	0.973	0.000	6	0.955	0.000	6	0.928	0.001
Condensed tannins	6	−0.952	0.000	6	−0.966	0.000	6	−0.901	0.002	6	−0.827	0.011
Total phenolics	6	−0.940	0.001	6	−0.936	0.001	6	−0.872	0.005	6	−0.802	0.017
SA ^a^	6	−0.908	0.002	6	−0.912	0.002	6	−0.853	0.007	6	−0.775	0.024
*PAL* ^b^	6	−0.963	0.000	6	−0.955	0.000	6	−0.909	0.002	6	−0.853	0.007
*PR1* ^c^	6	−0.966	0.000	6	−0.970	0.000	6	−0.943	0.000	6	−0.869	0.005

^a^ Salicylic acid; ^b^ Phenylalanine ammonia lyase; ^c^ Pathogenesis-related protein. “Weeks” refer to the start of the oviposition period of the single insect pairs (that occurred three weeks, four weeks and six weeks after the beginning of the experiment).

## Supplementary Materials

Supplementary materials can be found at www.mdpi.com/1422-0067/17/12/1964/s1.

## Abbreviations

TYLCV	*Tomato yellow leaf curl virus*
Wt	wild-type
JA	jasmonic acid
SA	salicylic acid
ppb	part per billion
ROS	reactive oxygen species
OTCs	open-top chambers
*PR1*	pathogenesis-related protein
*PAL*	phenylalanine ammonia lyase
*PI1*	proteinase inhibitor
*LOX*	lipoxygenase
*r_m_*	intrinsic rate of increase
TSWV	*Tomato spotted wilt virus*
AFLP	amplified fragment-length polymorphism
