# Supplementary material for: Elevated O3 and TYLCV Infection Reduce the Suitability of Tomato as a Host for the Whitefly Bemisia tabaci
Source: Int J Mol Sci. 2016 Nov 28;17(12):1964. doi: 10.3390/ijms17121964 (PMC5187764; doi:10.3390/ijms17121964)
Supplement: Supplementary file 1 [file ijms-17-01964-s001.pdf]

Supplementary Materials: Elevated O_3 and TYLCV Infection Reduce the Suitability of Tomato as a Host for the Whitefly *Bemisia tabaci*

Hongying Cui, Yucheng Sun, Fajun Chen, Youjun Zhang and Feng Ge

Table S1. Primer sequences used for real-time quantitative PCR.

Gene	Primer Sequence (5′-3′)	Fragment Length (bp)	Function
PI1	*F*: GCC AGA ACT TAT TGG TGT A<br>*R*: TGA CAT ATT GTG GCT GCT T	230	JA-defense related
LOX	*F*: ATG GCG ACA AGA AAG ATG AGG<br>*R*: CTT AAA GTA GGG CGA TTA GGG	163	JA-defense related
PAL	*F*: AGA TTG AAG TCA TTC GTG CTG<br>*R*: ACC CGT TGT TGT AAT AGT CGT TG	228	SA-defense related
PR1	*F*: TAC GCT ACC AAC CAA TGT G<br>*R*: TCC AGT TGC CTA CAG GAT C	151	SA-defense related
β-actin	*F*: GTT GGA ATG GGT CAG AAA GAT<br>*R*: TTC AGT AAG CAG AAC AGG GTG	189	Housekeeping gene
